# Supplementary figures and images for: Genome-Wide Identification of Freezing-Responsive Genes in a Rapeseed Line NTS57 Tolerant to Low-Temperature
Source: Int J Mol Sci. 2024 Nov 21;25(23):12491. doi: 10.3390/ijms252312491 (PMC11641804; doi:10.3390/ijms252312491)

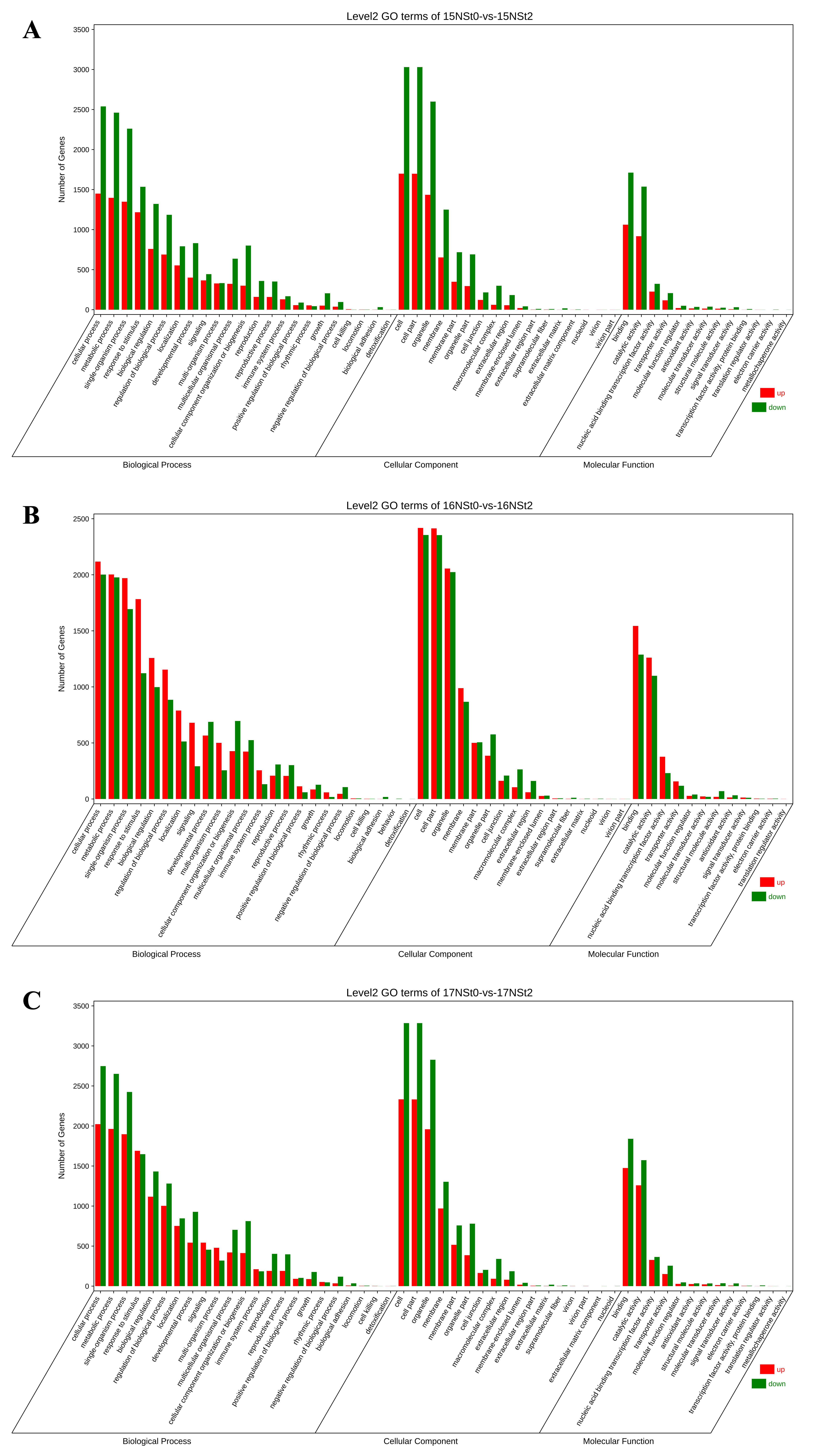

Supplement: Supplementary file 1 [file ijms-25-12491-s001.zip › Figure S1. GO classification analysis of the DEGs identified in 15NSt0-vs-15NSt2 (A), 16NSt0-vs-16NSt2 (B) and 17NSt0-vs-17NSt2 (C), respectively.jpg]

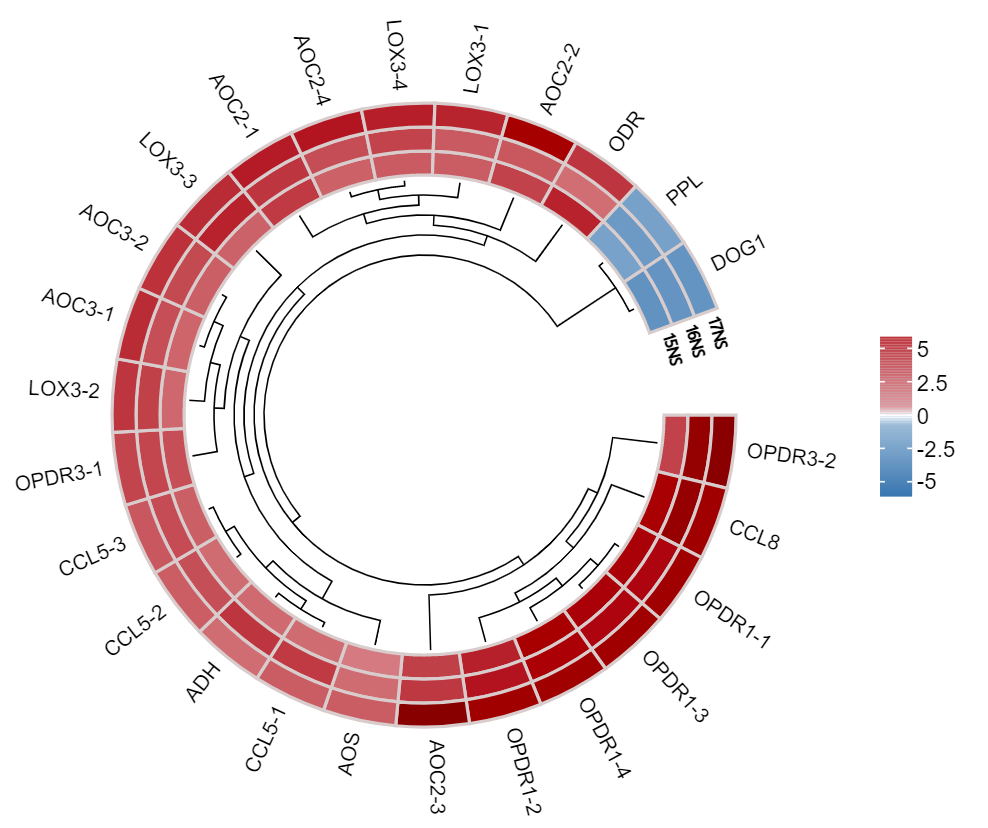

Supplement: Supplementary file 1 [file ijms-25-12491-s001.zip › Figure S2. Expression changes of DEGs enriched in alpha-linolenic acid metabolism pathway.jpg]

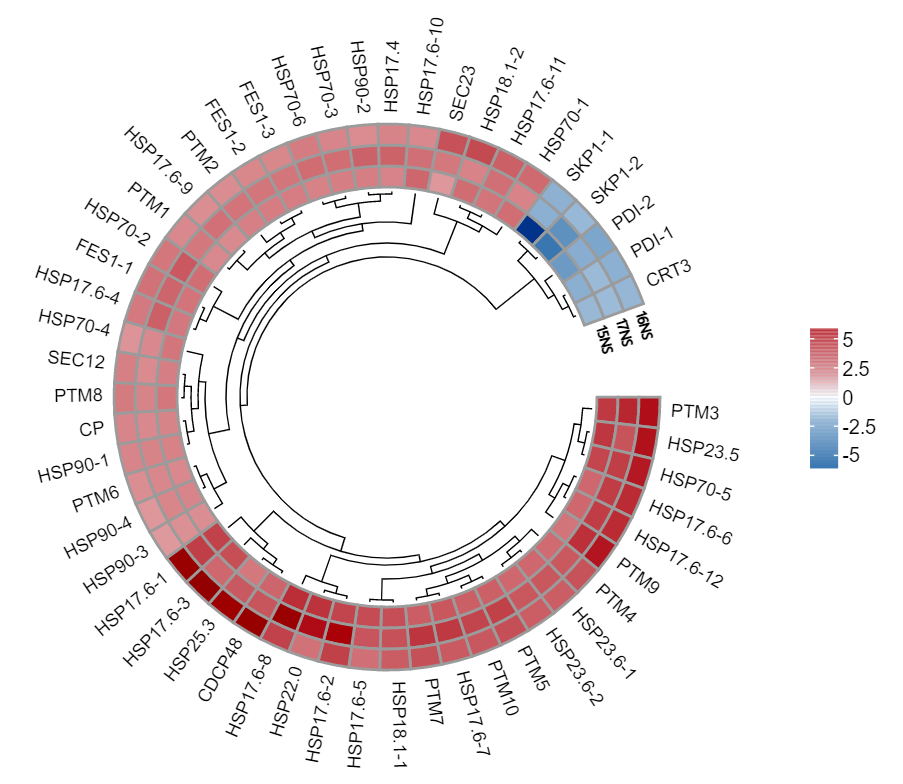

Supplement: Supplementary file 1 [file ijms-25-12491-s001.zip › Figure S3. Expression changes of DEGs enriched in protein processing metabolism pathway.jpg]

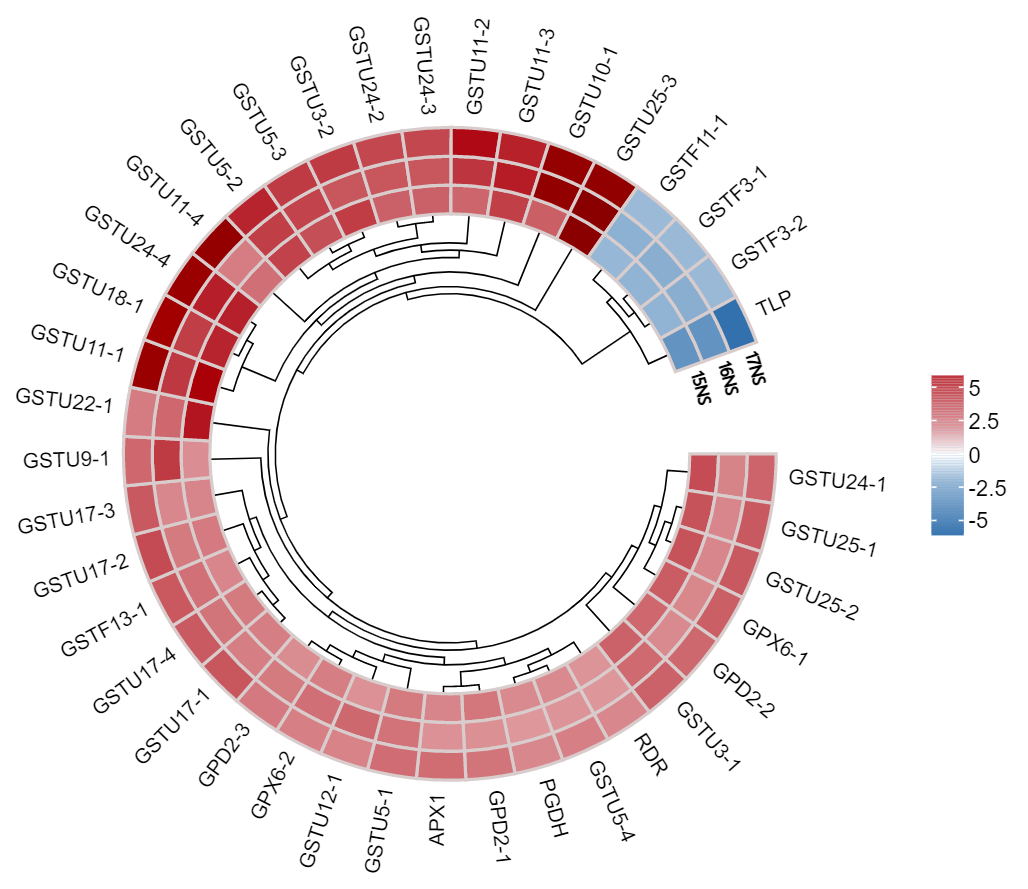

Supplement: Supplementary file 1 [file ijms-25-12491-s001.zip › Figure S4. Expression changes of DEGs enriched in glutathione metabolism pathway.jpg]

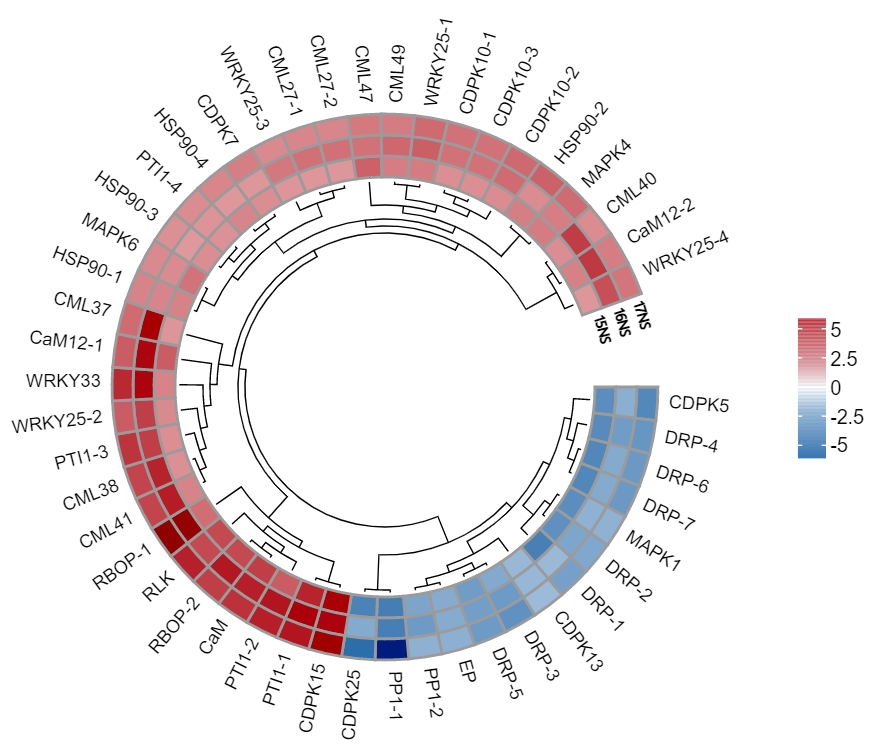

Supplement: Supplementary file 1 [file ijms-25-12491-s001.zip › Figure S5. Expression changes of DEGs enriched in plant pathogen interaction metabolism pathway.jpg]
